# Pan-Immune Inflammation Value in Detecting Perioperative Complications in Patients Undergoing Laparoscopic Sleeve Gastrectomy: A Prospective Cohort Study

**DOI:** 10.1007/s11695-026-08626-0

**Published:** 2026-03-27

**Authors:** Nizamettin KUTLUER, Ali AKSU, Mehmet Buğra BOZAN, Hakan AYYILDIZ, Ayşe AZAK BOZAN, Ali İŞLER, Ali ÖZKÖMEÇ, Mehmet Fatih EBİLOĞLU, Muhammed ALKAN, Onur AĞ, Burak GÜNEŞ, Cüneyt KIRKIL

**Affiliations:** 1 Department of Surgery, Turkish Ministry of Health, Medical Sciences University, Elazığ Fethi Sekin City Hospital, Elazığ, Turkey; 2 Department of Surgery, Elazığ Private Doğu Anadolu Hospital, Elazığ, Turkey; 3 Department of Surgery, Kahramanmaraş Sütçü İmam University, Kahramanmaraş, Turkey; 4 Department of Biochemistry, Turkish Ministry of Health, Medical Sciences University, Elazığ Fethi Sekin City Hospital, Elazığ, Turkey; 5 Department of Anesthesiology and Reanimation, Turkish Ministry of Health, Medical Sciences University, Elazığ Fethi Sekin City Hospital, Elazığ, Turkey; 6 Department of Surgery, Kahramanmaraş Sütçü İmam University, Kahramanmaraş, Turkey; 7 Department of Surgery, Balıklıgöl State Hospital, Şanlıurfa, Turkey; 8 Department of Surgery, Elazığ Private Medikal Hospital, Elazığ, Turkey

**Keywords:** Morbid Obesity, Laparoscopic Sleeve Gastrectomy, Clavien Dindo Surgical Complication Score, Perioperative Complication, PIV

## Abstract

**Objectives:**

Obesity is a significant health problem with increasing prevalence worldwide and is associated with numerous comorbidities. In this prospective controlled study, the relationship between changes in platelet-to-lymphocyte ratio(PLR), neutrophil-to-lymphocyte ratio(NLR), and pan-immune-inflammation value(PIV)-calculated from complete blood count parameters on the preoperative day and the first postoperative day-and early major surgical complications following laparoscopic sleeve gastrectomy(LSG) was investigated.

**Materials and Methods:**

Patients scheduled for LSG due to morbid obesity at the Department of General Surgery, Kahramanmaraş Sütçü İmam University, and Elazig Private Eastern Anatolia Hospital were prospectively included in the study. Preoperative and postoperative (24-hour) complete blood count (CBC) parameters were recorded, including white blood cell count (WBC), neutrophil count, lymphocyte count, monocyte count, and platelet count. PLR, NLR, and PIV were manually calculated. Patients were divided into two groups based on whether postoperative complications developed.

**Results:**

In the preoperative period, significant differences were observed in WBC count, neutrophil count, and PIV values between patients with and without complications (*p* = 0.001, *p* < 0.001, and *p* < 0.001, respectively). In the postoperative period, significant differences were found between the two groups in WBC count, neutrophil count, platelet count, PLR, NLR, and PIV (*p* = 0.001, *p* < 0.001, *p* < 0.001, *p* = 0.047, *p* = 0.001, and *p* < 0.001, respectively). When preoperative and postoperative values were compared, NLR, PLR, and PIV showed a statistically significant increase (*p* = 0.001, *p* < 0.001, and *p* < 0.001, respectively).

**Conclusion:**

Our study demonstrated that among inflammatory markers calculated from CBC parameters, the PIV-assessing the overall immune-inflammatory response-provides higher specificity and sensitivity than NLR and PLR in predicting early postoperative major complications following LSG.

**Supplementary Information:**

The online version contains supplementary material available at 10.1007/s11695-026-08626-0.

## Keypoints

The primary keypoint to assess whether the changes in preoperative and postoperative PIV, NLR, and PLR values could predict early major postoperative complications following LSG.

The Secondary keypoint included evaluation of perioperative major complication rates, reoperation rates, and mortality rates.

**Supplementary Information:**

The online version contains supplementary material available at 10.1007/s11695-026-08626-0.

## Introduction

Obesity is a significant health concern with increasing prevalence and is accompanied by numerous comorbidities [1]. In 2022, 2.5 billion adults (aged 18 and over) were classified as overweight (25 kg/m2 ≤ BMI < 30 kg/m2), and among these, 890 million were obese (BMI ≥ 30 kg/m2) [2].

Bariatric surgery is the most effective treatment modality for severe obesity [3, 4]. Bariatric and metabolic surgical procedures are performed even in patients with type-III obesity or higher who do not have any additional associated medical problems or type II severe obese patients with associated medical problems [4]. Thus, a variety of bariatric metabolic surgical techniques have been developed. Currently, laparoscopic sleeve gastrectomy (LSG) and Roux-en-Y gastric bypass are the most commonly performed bariatric metabolic procedures [5, 6]. However, each surgical procedure inevitably entails the risk of complications. Among the short-term perioperative complications, bleeding, staple line leaks, deep venous thrombosis, and pulmonary thromboembolism are particularly significant [7, 8]. These complications contribute to increased hospital readmissions, mortality, and the necessity for reoperations.

The development of complications triggers an increase in inflammatory activity in the body. Complete blood count (CBC) parameters and various ratios derived from these parameters are used to assess the body’s inflammatory status [9, 10]. These parameters are utilized in evaluating postoperative complications due to their low cost and ease of calculation [9]. The neutrophil-to-lymphocyte ratio (NLR) and platelet-to-lymphocyte ratio (PLR) have been previously shown to aid in detecting complications following gastric surgeries [9, 10]. Similarly, the pan-immune inflammation value (PIV), calculated from CBC parameters, is another factor used in evaluating the body’s inflammatory response [11].

This prospective cohort study aimed to investigate the relationship between changes in PIV, NLR, and PLR, calculated from CBC parameters obtained during the preoperative period and on the first postoperative day following LSG, and the occurrence of major surgical complications in the early postoperative period.

## Materials and Methods

After getting approval from the local ethics committee (The Medical Research Ethical Commitee of Kahramanmaras Sutcu Imam University, Date: 26.04.2022; Session Number: 2022/14; Decision No: 06), patients scheduled for surgery due to morbid obesity and undergoing LSG as the surgical procedure were prospectively enrolled at the General Surgery Department of Kahramanmaras Sutcu Imam University and General Surgery Clinic of Elazig Special Eastern Anatolia Hospital between June 1, 2022 and June 1, 2023. At the conclusion of the study, the data were retrospectively evaluated.

For patients undergoing LSG, routine preoperative CBC parameters and the parameters obtained 24 h postoperatively were measured using an automated hematology analyzer (XN 3000; Sysmex Corp., Kobe, Japan, and Beckman DxH800; Beckman Coulter, Inc., Miami, FL, USA) for quantifying white blood cell, neutrophil, lymphocyte, monocyte, and platelet counts. Manually, the PLR [Platelet Count (109/L): Lymphocyte Count (109/L)], NLR [Neutrophil Count (109/L): Lymphocyte Count (109/L)], and PIV [Neutrophil Count (109/L) × Platelet Count (109/L) × Monocyte Count (109/L): Lymphocyte Count (109/L)] were calculated. During follow-up, patients were categorized into two groups: Group 1 comprised patients undergoing LSG for severe obesity without perioperative complications, whereas Group 2 included patients undergoing LSG for severe obesity with perioperative complications of Grade III, IV, or V according to the Clavien–Dindo classification [12]. This study adhered to the Standards for the Reporting of Diagnostic Accuracy Studies (STARD) reporting guidelines for cohort studies [13]. All procedures performed in studies involving human participants were in accordance with the ethical standards of the institutional and/or national research committee and with the 1964 Declaration of Helsinki, as well as its subsequent amendments or comparable ethical standards.

### Inclusion Criteria

1. Patients aged > 18 and < 65 years
2. Patients who consented to participate in the study
3. Patients with an elective surgical indication due to severe obesity and scheduled for LSG as the primary operation (patients with a BMI $\:\ge\:$40 kg/m^2^ and without any known associated medical problems)
4. Patients with complications classified as Grade III or higher according to the Clavien–Dindo classification

### Exclusion Criteria

1. Patients with severe obesity aged < 18 or > 65 years
2. Patients with an elective surgical indication due to severe obesity and scheduled for a bariatric surgery other than LSG as the primary operation
  - Patients with a BMI of $\:\ge\:$40 kg/m^2^ and no known associated medical problems
  - Patients with a BMI of $\:\ge\:$35 kg/m^2^ who had associated medical problems (e.g., diabetes, hypertension, asthma, chronic obstructive pulmonary disease)
3. Patients scheduled for revisional surgery.
  - Patients scheduled for revisional surgery due to weight regain.
  - Patients with chronic complications resulting from the initial bariatric surgery (e.g., esophagitis, stenosis, marginal ulcer, malnutrition).
4. Patients unwilling to participate in the study.
5. Patients with concomitant conditions, such as malignancies or rheumatologic diseases, that could alter inflammatory parameters.
6. Patients with complications classified as Grade I or II according to the Clavien–Dindo classification [12].

The sample size was calculated using the G*Power 3.10 program, resulting in a total of 112 patients categorized into two groups, with an α level of 0.05, a power (1-β) of 0.95, and an effect size of 0.3.

### Surgical Technique

During preoperative preparation, patients received low molecular weight heparin (0.6 cc enoxaparin) 12 h before surgery to reduce the risk of deep vein thrombosis.

Surgery was performed using our preferred sleeve gastrectomy technique, using four incisions and inserting one camera trocar, two surgical procedure trocars, and one automatic liver retractor. The stomach was then freed from the omentum along the greater curvature using energy devices. A 36-French gastric calibration tube was inserted into the stomach. The stomach was removed by taking the fundus, starting approximately 3 cm from the pylorus and focusing more on the fundus, 1 cm from the angle of Hiss, using staplers with tristaple technology. An average of five manual staplers was used per patient. Visible fat pads were routinely removed. Routine intraoperative leak testing was performed using methylene blue. Stapler line reinforcement procedures (such as suturing or omentopexy) were not routinely performed unless the leak test revealed suspicion. A clip was routinely applied along the staple line to control bleeding. A drain was placed along the staple line, extending into the subdiaphragmatic space.

### Statistical Analysis

Statistical analysis was performed using the IBM Statistical Package for Social Sciences (SPSS) version 20.0. Categorical data were presented as numbers (n) and percentages (%), whereas continuous variables were expressed as median (25th–75th percentiles) or median (95% Confidence Interval). The normality of continuous variables was assessed using the Shapiro–Wilk test along with post hoc analysis. For comparisons between groups, the Mann–Whitney U test was used for continuous variables, and the chi-square test was used for categorical variables. The repeated measures linear model was used to evaluate differences in repeated measurements. A p-value of < 0.05 was considered statistically significant in all analyses.

### Outcomes

The primary outcome of the study was to assess whether the changes in preoperative and postoperative PIV, NLR, and PLR values could predict early major postoperative complications following LSG. Secondary outcomes included evaluation of perioperative major complication rates, readmission rates, reoperations, and mortality.

## Results

**Fig. 1:**
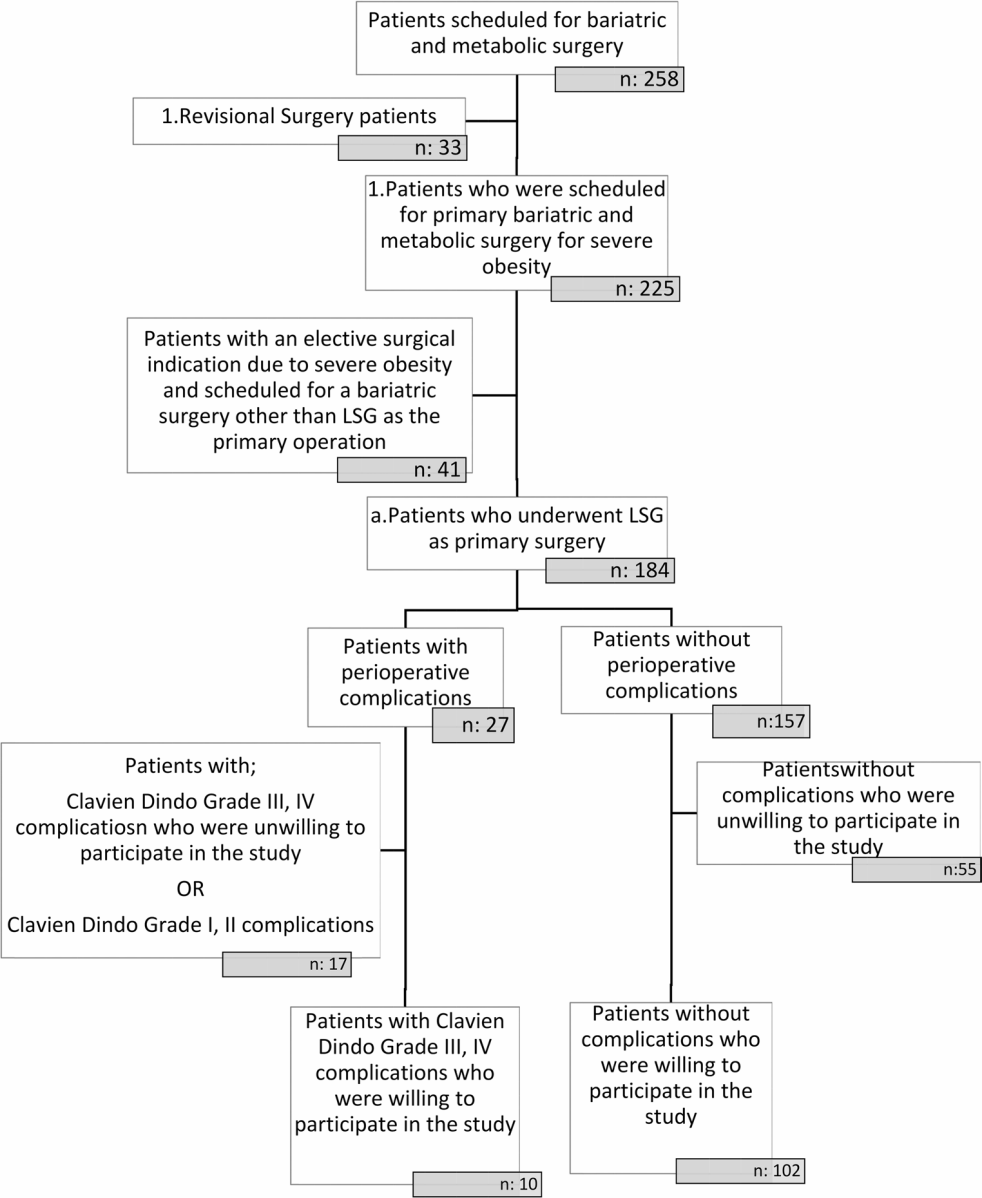
Flowchart of the Study Design (LSG: Laparoscopic Sleeve Gastrectomy)

During the study period, 112 patients who met the inclusion criteria and consented to participate were evaluated. Among these, 10 patients (8.9%) developed major perioperative complications according to the Clavien–Dindo classification, whereas 102 patients (91.1%) did not experience any perioperative complications (Fig. 1). Among the patients with major complications, three developed staple line leakage (2.68%), two experienced pulmonary thromboembolism (1.79%), and five developed intra-abdominal hemorrhage (comprising three cases of trocar site bleeding and two cases of staple line bleeding) (4.46%). The leakage was identified on the fourth day after surgery, with routinely applied radiologic evaluations. The bleeding was seen after the first day of the surgical intervention. The pulmonary thromboembolism cases were seen on the second and third days after surgery. Reoperation was required in three patients with complications (one case of staple line leakage and two cases of intra-abdominal hemorrhage). No mortality was observed.

When comparing patients with and without complications, no statistically significant differences were observed in terms of sex, type of obesity, age, height, weight, or BMI (*p* > 0.05 for all variables) (Table 1).

**Table 1:** Demographic Parameters for Study Groups

		Perioperative Complication		*p* value
		Yes	No	
Sex	Female (n%)	4 (3,6%)	69 (61,6%)	0.83
	Male (n%)	6 (5,4%)	33 (29,5%)	
Total (n%)		10 (8,9%)	102 (91,1%)	
Obesity Type	Type III	8 (7.1%)	86 (76.8%)	0.806
	Type IV	2 (1.8%)	14 (12.5%)	
	Type V	0 (0%)	2 (1.8%)	
Total (n%)		10 (8.9%)	102 (91.1%)	
Age (years) Median (%25 - %75 persentile)		29 (20–38)	32 (24–41)	0.435
Height (cm) Median (%25 - %75 persentile)		168 (161.81–175.99)	166 (164.98–168.66)	0.592
Weight (kg) Median (%25 - %75 persentile)		130 (114.99–144.41)	121 (120.56–128.75)	0.414
BMI (kg/m^2^) Median (%25 - %75 persentile)		43.78 (43.54–50.06)	42.66 (40.60–45.81)	0.838

*BMI* Body Mass Index

*p < 0.05 according to the Mann-Whitney U test analysis

**p < 0.05 according to the x^2^ analysis

In the postoperative period, statistically significant differences were observed in WBC count, neutrophil count, platelet count, PLR values, NLR values, and PIV values between patients with and without complications (p-values of = 0.001, < 0.001, < 0.001, = 0.047, = 0.001, and < 0.001, respectively). Furthermore, no significant differences were found in the remaining CBC parameters between these groups (*p* > 0.05) (Table 2).

**Table 2:** Parametric Parameters Between Study Groups

	Perioperative Complication		*p* value
	Yes Median (%95 Confidence Interval)	No Median (%95 Confidence Interval)	
Preoperative WBC (10^9^/L)	11.05 (10.08–11.85)	9 (8.86–9.51)	0.001*
Preoperative Neutrophyl count (10^9^/L)	8.66 (7.67–9.50)	5.57 (5.54–6.17)	< 0.001*
Preoperative Lymphocyte Count (10^9^/L)	3.74 (1.998–3.87)	2.46 (2.43–2.69)	0.27
Preoperative Monocyte Count (10^9^/L)	0.63 (0.54–0.76)	0.53 (0.51–0.58)	0.073
Preoperative Platelet Count (10^9^/L)	325 (268.66–386.94)	285 (279.58–304.46)	0.209
Postoperative WBC (10^9^/L)	19.75 (14.36–22.45)	11.8 (9.69–10.97)	0.001*
Postoperative Neutrophyl Count (10^9^/L)	18.065 (14.86–19.99)	10.07 (8.14–12.05)	< 0.001*
Postoperative Lymphocyte Count (10^9^/L)	1.55 (0.94–2.65)	1.69 (1.66–2.57)	0.366
Postoperative Monocyte Count (10^9^/L)	1.045 (0.799–1.29)	0.815 (0.78–0.9)	0.72
Postoperative Platelet Count (10^9^/L)	350.5 (301.43–377.37)	264 (253.53–274.12)	< 0.001*
Preoperative NLR	2.58 (2.32–4.93)	2.28 (2.25–2.65)	0.029*
Preoperative PLR	107.41 (77.22–211.09)	108.39 (113.25–130.65)	0.947
Preoperative PIV	695.19 (454.75–990.43)	335.91 (336.43–424.65)	< 0.001*
Postoperative NLR	11.79 (7.496–18.61)	5.82 (5.75–7.07)	0.001*
Postoperative PLR	195.93 (79.47–678. 57)	152.68 (146.06–171.42)	0.047*
Postoperative PIV	4540.28 (2855.57–5882.72)	1237.57 (1254.54–1647.51)	< 0.001*

*PIV* Panimmune Inflammation Value, *WBC* White Blood Cell Count, *NLR* Neutrophil to lymphocyte ratio, *PLR* Platelet to lymphocyte ratio

*p < 0.05 according to the Mann-Whitney U test

**Fig. 2:**
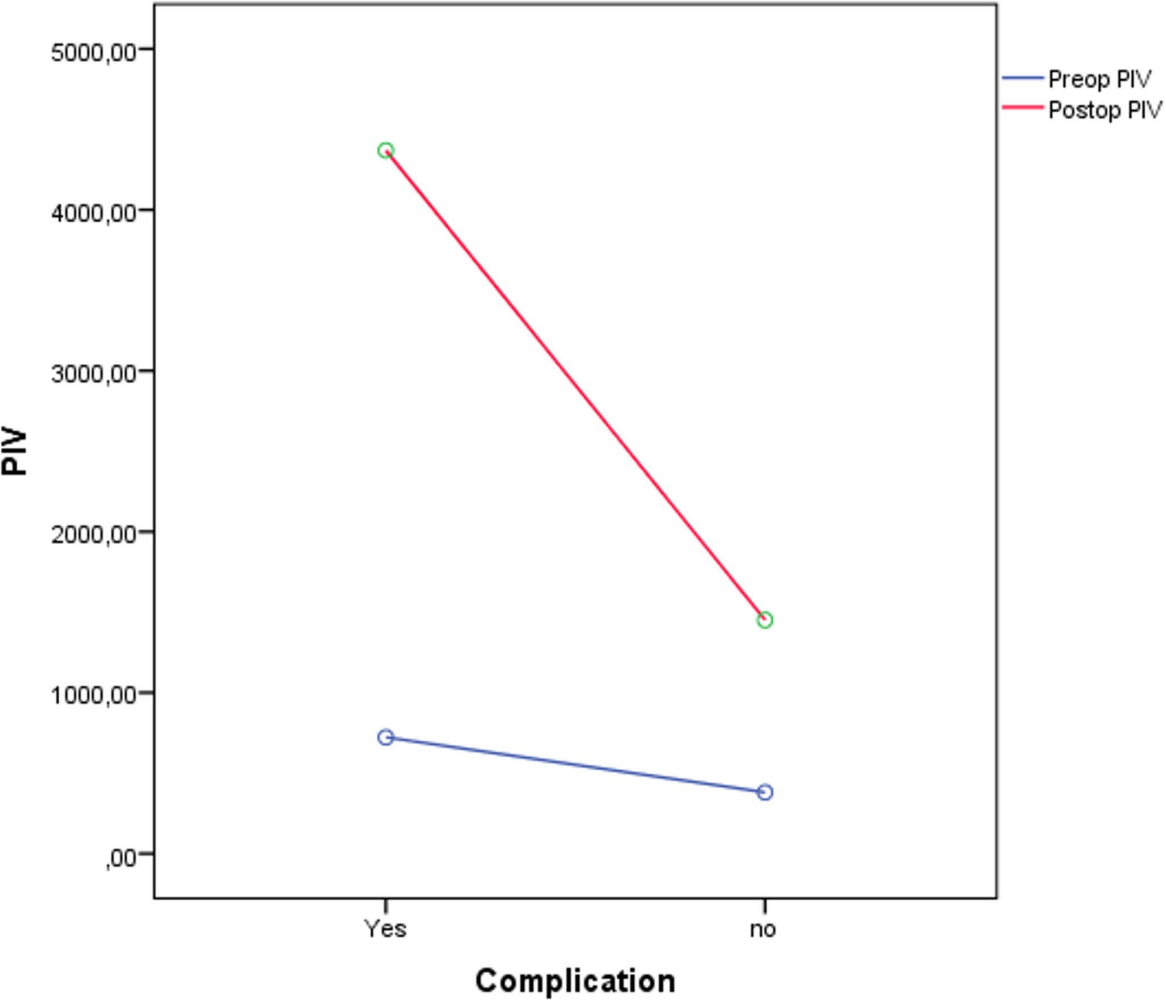
Preoperative and Postoperative PIV Results Between Groups

When the changes between preoperative and postoperative NLR values were examined, the increase in the number of patients with complications was significantly greater than that in patients without complications (F = 10.653, *p* = 0.001, mean2 = 32698.495, ꬼ2 ≈ 0.214). Similarly, the changes between preoperative and postoperative PLR values revealed that the increase in the number of patients with complications was significantly different from that in patients without complications (F: 11.424, *p* = 0.001, mean2: 74199.603, ꬼ2 ≈ 0.094). Moreover, the increase in PIV values between the preoperative and postoperative periods was significantly greater in patients who developed complications than those who did not (F: 62.995, *p* < 0.001, mean2 = 48398476.186, ꬼ2 ≈ 0.364) (Fig. 2).

**Fig. 3:**
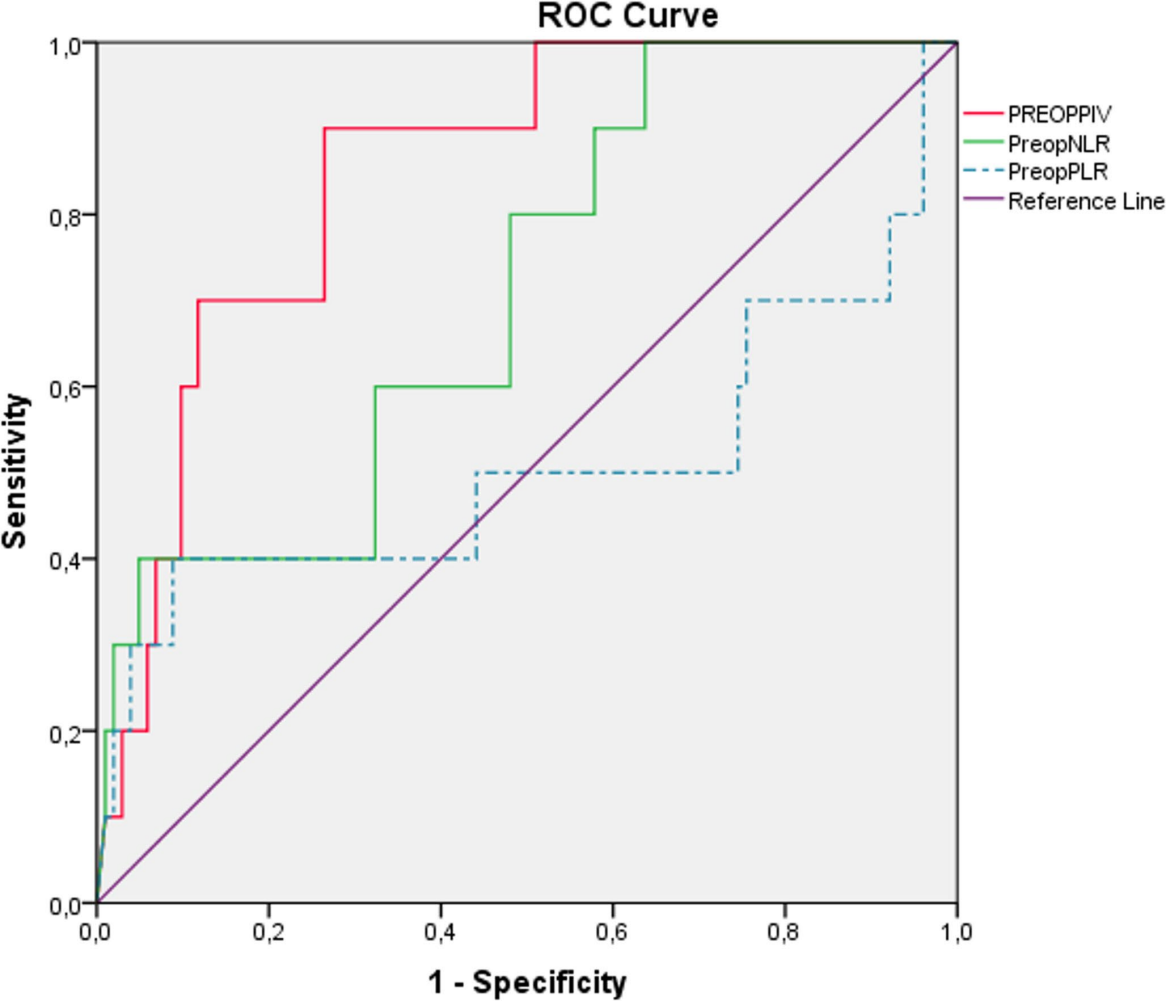
ROC Analysis of Preoperative PIV, NLR and PLR Values

ROC curve analysis of preoperative NLR, PLR, and PIV values for predicting complications in severe obese patients: In patients undergoing primary LSG for morbid obesity, the preoperative evaluation for predicting perioperative complications revealed that the optimal cut-off value for NLR was ≥ 2.43, with a sensitivity of 60% and a specificity of 60.8% (AUC: 0.709; 95% CI: 0.551–0.867). For PLR, the cut-off value was ≥ 119.34, yielding a sensitivity of 60% and a specificity of 60.8% (AUC: 0.506; 95% CI: 0.257–0.756). For PIV, the cut-off value was ≥ 501.71, with a sensitivity of 70% and a specificity of 83.5% (AUC: 0,849; 95% CI: 0.784–0.911) (Fig. 3; Table 3).

**Table 3:** ROC Analysis of Preoperative PIV, NLR and PLR Values Between Groups

	AUC	Cut-off value	Specificity (%)	Sensitivity (%)	95% CI		*p* value
					Lower Bound	Upper Bound	
Preoperative PIV	0.849	501,71	70	83.5	0.746	0.951	< 0.001*
Preoperative NLR	0.709	2.43	60	60.8	0.551	0.867	< 0.029*
Preoperative PLR	0.506	119.34	50	55.9	0.257	0.756	0.947

*PIV* Panimmune Inflammation Value, *NLR* Neutrophil to lymphocyte ratio, *PLR* Platelet to lymphocute ratio, *AUC* Area Under Curve, *CI* Confidence Interval

*p < 0.05

**Fig. 4:**
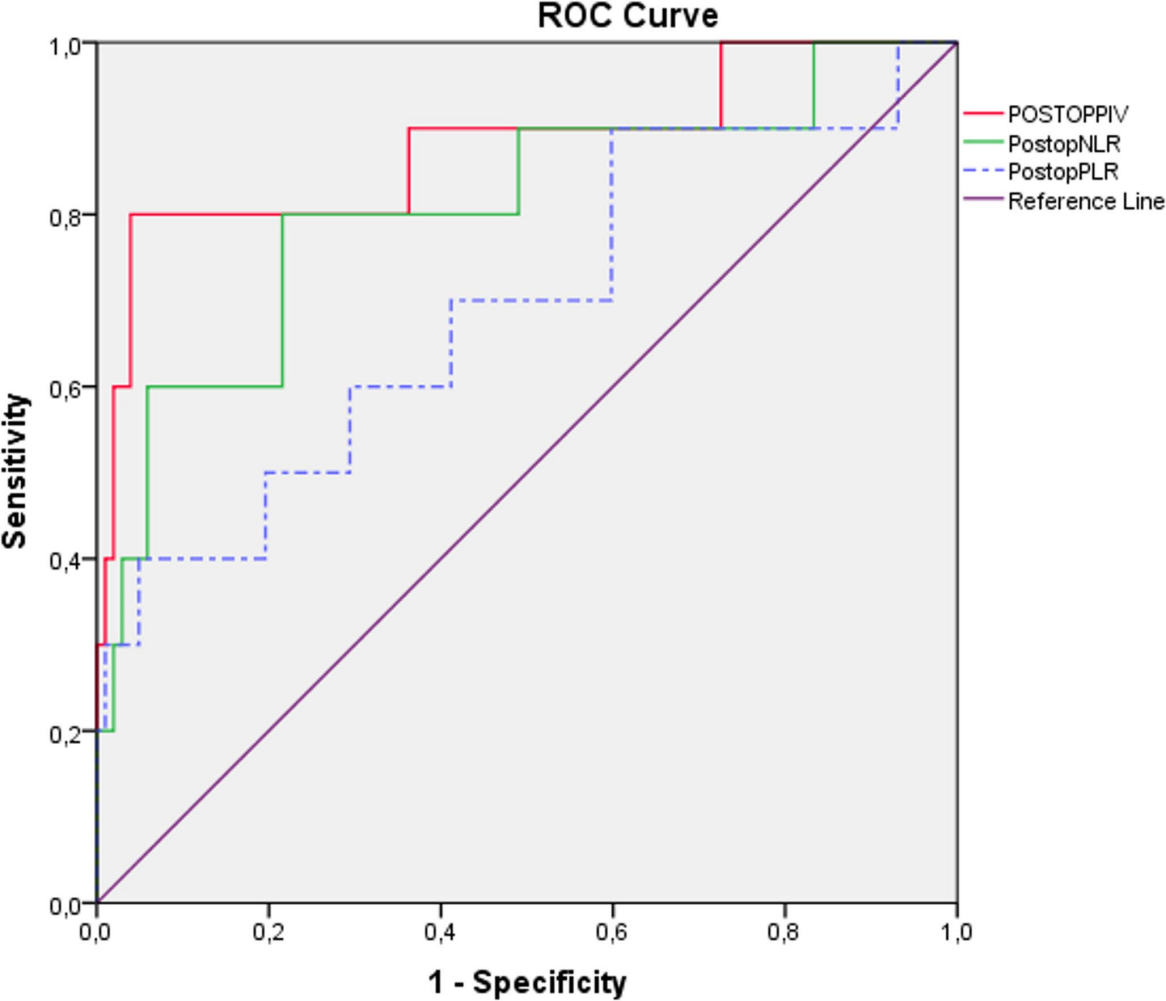
ROC Analysis of Postoperative PIV, NLR, and PLR Values

ROC curve analysis of postoperative NLR, PLR, and PIV values for predicting complications in severe obese patients: Regarding the postoperative evaluation on the first day following surgery, the ROC curve analysis demonstrated that the optimal cut-off value for NLR was ≥ 8.77, with a sensitivity of 80% and a specificity of 78.4% (AUC: 0.808; 95% CI: 0.644–0.972). For PLR, the cut-off value was ≥ 179.59, with a sensitivity of 60% and a specificity of 70.6% (AUC: 0.691; 95% CI: 0.497–0.885). For PIV, the cut-off value was ≥ 2717.44, yielding a sensitivity of 80% and a specificity of 91.2% (AUC: 0,878; 95% CI: 0.706–1.000) (Fig. 4; Table 4).

**Table 4:** ROC Analysis of Postoperative PIV, NLR and PLR Values Between Groups

	AUC	Cut-off value	Spesificity (%)	Sensitivity (%)	95% CI		*p* value
					Lower Bound	Upper Bound	
PostoperativePIV	0.878	2717.44	80	91.2	0.706	1.000	< 0.001*
Postoperative NLR	0.808	8.77	80	78.4	0.644	0.972	0.001*
Postoperative PLR	0.691	179.59	60	70.6	0.497	0.885	0.047*

*PIV* Panimmune Inflammation Value, *NLR* Neutrophil to lymphocyte ratio, *PLR* Platelet to lymphocyte ratio, *AUC* Area Under Curve, *CI* Confidence Interval

*p < 0.05

After univariate, multivariate, and binary logistic regression analysis performed in terms of factors affecting the development of complications, preoperative WBC, neutrophil count, preoperative PIV, and preoperative NLR values ​​were found to be predictive factors for the complication (Table 5).

**Table 5:** Multivariate Analysis of Preop CBC Parameters and CBC-Derived Parameters

	B Score	T Score	*p* value	95% Confidence Interval		OR	F Score
				Lower Bound	Upper Bound		
Age	-2.218	-0.666	0.507	-8.818	4.383	0.004	0.443
Preop WBC	1.778	3.320	< 0.001	0.717	2.840	0.091	11,023
Preop Neutrophil Count	2.731	5.234	< 0.001	1.697	3.764	0.199	27,399
Preop Lymphocyte Count	0.373	1.502	0.136	-0.119	0.865	0.020	2,255
Preop Platelet Count	35.780	1.658	0.100	-6.989	78.550	0.024	2,749
Preop PIV	342.048	4.295	< 0.001	184.205	499.891	0.144	18,443
Preop NLR	1.169	3.241	0.002	0.454	1.884	0.087	,132
Preop PLR	22.204	1.335	0.185	-10.751	55.159	0.016	10,507
Preop BMI	0.694	0.363	0.717	-3.088	4.475	0.001	1,783

## Discussion

Obesity and obesity-related health problems are increasing in Turkey, similar to the global scenario. In Turkey, the average BMI increased from 33.1 kg/m2 in 2020 to 34.3 kg/m2 in 2022 [14]. Moreover, the obesity prevalence in Turkey was 35.6% in 2022, with 20% of these cases classified as Type III or higher conditions that typically warrant surgical intervention [15]. Due to this increase, bariatric surgical procedures have emerged as a forefront treatment for health problems caused by obesity [1, 3].

Since biliopancreatic diversion with duodenal switch was defined as the first-step treatment, LSG has become the most commonly performed surgical procedure worldwide for severe obesity, with application rates reaching up to 50% [16]. The most frequent complications observed in the perioperative period following LSG, as also noted in the present study, include staple line leaks (which may be observed in 1%–3% after primary surgeries and up to 10% after revision surgeries), bleeding (1–6%), pulmonary thromboembolism (0.5%), venous thrombosis (1%), and surgical site infections (3%). Perioperative mortality rates remain below 1% [1, 17–19]. Boeker et al. show a 4.9% leakage rate as a result of being a teaching center. They addressed that the learning curve of junior surgeons could be the reason for the higher training curve [20]. Our major complication rates are similar to the literature (three developed staple line leakage (2.68%), two pulmonary thromboembolism (1.79%), and five intra-abdominal hemorrhage (4.46%)). Reoperation was required in three patients with complications (one case of staple line leakage and two cases of intra-abdominal hemorrhage). No mortality was observed. For diminishing the complication rates, omentopexy is advised in LSG cases [21, 22]. Otherwise, some studies show no differences between omentopexy and clipping [23]. Our slightly higher complication rates may recommend us to consider diminishing complication rates; stapline reinforcements, such as omentopexy, can be helpful. Additionally, one of our study centers is a teaching center, and like Boeker et al., our slightly higher rate of leakage and hemorrhage was elevated in the junior surgeon’s educational period [20].

It is crucial to predict the occurrence of life-threatening complications, such as staple line leaks, pulmonary thromboembolism, venous thrombosis, and bleeding, which may require medical or re-interventional management, and to enable early medical intervention. Thus, CBC parameters and the ratios derived from them, which are easily obtainable, inexpensive, and rapid, have been utilized to predict morbidity and mortality in various diseases, including bariatric surgery [10, 18, 19]. In a study conducted by Romano et al., NLR and procalcitonin levels were found to be effective in predicting leaks after LSG [24]. Similarly, in a study by Seyit et al., the NLR calculated from the CBC parameters on postoperative day 1 was demonstrated to be a useful marker in predicting postoperative leak development [25]. In another study by Makal and Yıldırım, the preoperative values and the postoperative day 1 and day 3 values of C-reactive protein/albumin ratio (CAR), NLR, and PLR were evaluated in predicting postoperative complications after LSG, and a difference was observed between patients with and without complications in all measurements for CAR and only on postoperative day 3 for PLR [26]. In contrast, Dinçer and Doğan found that preoperative NLR values did not predict the development of complications in a study of 95 patients undergoing mini gastric bypass [10]. Consistent with the findings of Romano and Seyit, the present study showed that NLR is effective in predicting complications following LSG. Contrary to the findings reported by Dinçer and Doğan as well as Makal and Yıldırım, the present study demonstrated that both the preoperative NLR value and the change in NLR on postoperative day 1 were significantly higher in patients who developed major complications. Furthermore, unlike Makal and Yıldırım, we found that the preoperative and postoperative day 1 PLR values, as well as the change in PLR, were significantly different in patients with major perioperative complications. We believe that this discrepancy may be due to the exclusion of patients with comorbid conditions, such as diabetes and chronic obstructive pulmonary disease, which could induce an inflammatory response, from our study cohort.

PIV, which is calculated from CBC parameters and allows for the simultaneous evaluation of all inflammatory cells (lymphocytes, monocytes, neutrophils, and platelets), is an important marker that reflects the systemic inflammatory response [11, 27, 28]. It has been used to predict prognosis in various malignancies such as colorectal and prostate cancers [11]. In studies by Mete Yıldırım and Yıldırım, PIV values were shown to assist in determining disease severity in patients with ulcerative colitis [27]. In a study by Yu et al., PIV was found to be an essential parameter in predicting postoperative in-hospital mortality in patients undergoing surgery for acute type A aortic dissection [11]. Similarly, Vural et al. observed that PIV values were proper in predicting postoperative mortality in patients with acute cholecystitis [28]. Furthermore, Abdul-Quddus et al. reported that increased PIV values were associated with obesity-related mortality [29]. In the present study, we also determined that the PIV values calculated during the preoperative period and on postoperative day 1, as well as the changes occurring between these two time points, helped predict perioperative complications following LSG. Our study showed that an increase of approximately 5 times or more in preoperative and postoperative PIV values ​​should suggest that the patient has perioperative complications.

### Limitations

The relatively small sample size of complicated cases in the present study is a significant limitation for the statistical analysis. However, the prospective follow-up of patients and the fact that this is the first study to investigate changes in PIV values in predicting major complications following LSG are notable strengths. In addition, the presence of patients with exclusion criteria and comorbidities (e.g., malignancies or rheumatological diseases) in the sample is another limitation of our study. Additionally, we didn’t use the C-reactive protein (CRP), eritrocyte sedimentation rate (ESR), and procalcitonin levels for the follow-up period routinely. This status is another limitation of our study. Additionally, we excluded minor complication cases (Clavien Dindo I-II), and our study’s protocol limitation consists of either patients without complications or with major complications. Being a dual-center study, our CBC calculations have two different analyzers. This is another limitation of our study. Although being a dual-center study may seem like an advantage, supporting it with more comprehensive studies will further strengthen our study.

## Conclusion

Assessing the inflammatory response through CBC parameters is a simple and cost-effective method. This prospective study has demonstrated that the PIV values, calculated from CBC parameters obtained during the preoperative period and on postoperative day 1, are significantly more sensitive and specific than NLR and PLR values in predicting major perioperative complications following LSG. Larger-scale studies that use other inflammation parameters in the follow-up period are necessary to support these findings, which may serve as an essential guide for bariatric metabolic surgeons in the early detection of increased risk of complications in patients.

## Supplementary Information

Below is the link to the electronic supplementary material.

Supplementary Material 1.

## Data Availability

No datasets were generated or analysed during the current study.

## Declarations

### Clinicaltrials Number

NCT06284356.
