# Supplementary material for: Pan-Immune Inflammation Value in Detecting Perioperative Complications in Patients Undergoing Laparoscopic Sleeve Gastrectomy: A Prospective Cohort Study
Source: Obes Surg. 2026 Mar 27;36(5):2349–60. doi: 10.1007/s11695-026-08626-0 (PMC13222289; doi:10.1007/s11695-026-08626-0)
Supplement: Supplementary file 1 — Supplementary Material 1. [file 11695_2026_8626_MOESM1_ESM.pdf]

Kahramanmaraş Sütçü İmam Üniversitesi Tıbbi Araştırmalar Etik Kurulu	
**Başvuru Bilgileri**	
**Araştırmanın Başlığı**	Laparoskopik Sleeve Gastrektomi Uygulanan Hastaların Perioperatif Komplikasyonlarının Tespitinde Pan İmmun İnflamasyon Değerleri: Prospektif Kohort Çalışması
**Sorumlu Araştırmacı**	Dr. Öğr. Üyesi Mehmet Buğra BOZAN
**Yardımcı araştırmacılar**	Opr.Dr. Nizamettin KUTLUER, Opr.Dr. Ali AKSU
**Başvuru Tarihi ve Protokol No**	07.03.2022 - 105

Karar Bilgileri<br>(Tüm üyelerin imzalarının bulunduğu nihai karar evrakı daha sonra verilecektir)			
**Oturum No**	2022/14	**Karar No**	06
**Tarih**	26.04.2022	**Karar**	OLUMLU

Başkan veya Başkan Yardımcısının İmzaları		
	**Unvanı/Adı/Soyadı**	**İmza**
**Başkan**	Prof. Dr. Yusuf Ergün	
